# Superhydrophobic Natural and Artificial Surfaces—A Structural Approach

**DOI:** 10.3390/ma11050866

**Published:** 2018-05-22

**Authors:** Roxana-Elena Avrămescu, Mihaela Violeta Ghica, Cristina Dinu-Pîrvu, Răzvan Prisada, Lăcrămioara Popa

**Affiliations:** Department of Physical and Colloidal Chemistry, Faculty of Pharmacy, University of Medicine and Pharmacy ”Carol Davila”, Bucharest 020956, Romania; roxana.avramescu@drd.umfcd.ro (R.-E.A.); ecristinaparvu@yahoo.com (C.D.-P.); razvan.prisada@gmail.com (R.P.); lacramioara.popa@umfcd.ro (L.P.)

**Keywords:** special surfaces, wettability, superhydrophobic, cell cultures, anti-bio adhesion, self-cleaning fabrics

## Abstract

Since ancient times humans observed animal and plants features and tried to adapt them according to their own needs. Biomimetics represents the foundation of many inventions from various fields: From transportation devices (helicopter, airplane, submarine) and flying techniques, to sports’ wear industry (swimming suits, scuba diving gear, Velcro closure system), bullet proof vests made from Kevlar etc. It is true that nature provides numerous noteworthy models (shark skin, spider web, lotus leaves), referring both to the plant and animal kingdom. This review paper summarizes a few of “nature’s interventions” in human evolution, regarding understanding of surface wettability and development of innovative special surfaces. Empirical models are described in order to reveal the science behind special wettable surfaces (superhydrophobic /superhydrophilic). Materials and methods used in order to artificially obtain special wettable surfaces are described in correlation with plants’ and animals’ unique features. Emphasis is placed on joining superhydrophobic and superhydrophilic surfaces, with important applications in cell culturing, microorganism isolation/separation and molecule screening techniques. Bio-inspired wettability is presented as a constitutive part of traditional devices/systems, intended to improve their characteristics and extend performances.

## 1. Introduction

Over the last decades, surface and interfacial phenomena gained interest among researchers, especially through applications which ease medical or industrial procedures, giving the latter the attribute of being “environmental friendly”. The present paper focuses on the progress made in understanding and discovering new superficial properties of certain surfaces. The journey into the micronical size world begins with a short introduction into basic surface chemistry elements, in relation with the chemical structure of solids. Understanding surface phenomena makes it easier to unravel some yet unexplained superficial behavior and represents the starting point in developing useful applications for human kind. A detailed description of surfaces encountered in the natural (vegetal/animal) environment, is followed by a summary of methods used to obtain solid supports exhibiting special surface properties. Fast development in wettable surface engineering led to the discovery of novel applications in medical fields (biomolecule monitoring, cancer cell isolation), transportation, cleaning and other industries.

## 2. Superficial Properties

### 2.1. Special Wettable Surfaces

Surface wettability characterizes the interfacial phenomena between a liquid and a solid support. The liquid’s behavior is in fact the wettability indicator. This superficial property is studied in order to establish hydrophilicity/hydrophobicity of a solid, offering an open gate to numerous every-day life applications (self-cleaning/anti-bacterial fabrics, anti-reflection, transparent coatings) and also industrial ones (anti-icing surfaces, special surface patterns). Superhydrophobic surfaces which display a contact angle higher than 150°, or 180°, (according to other authors) and a sliding angle smaller than 10° attracted attention of researchers. They were first observed in the natural environment (Lotus leave, butterfly wings, fish scales etc.). Following the principles of biomimetics, special superficial surface properties were adapted to human necessities and used as a model in many industrial areas, including nanotechnologies. In time, many applications related to superhydrophobicity were unraveled: Development of self-cleaning and low friction surfaces, satellite antennas, solar and photovoltaic panels, exterior glass, swimming suits etc. Studies show that these superhydrophobic surfaces have many other attributes: They prevent bacteria adhesion, metal corrosion, improve blood type compatibility, lower surface icing in humid atmosphere and low temperature conditions, are constitutive parts of water storage systems and of microreactors, in which new reaction compounds are produced [1].

Apart from superhydrophobic surfaces, superoleophobic ones gain researchers’ attention. The suffix “oleo-” refers to liquids of low-surface tension (oils) and other organic liquids. Superoleophobicity represents the wetting phenomenon characterized through oil droplets displaying low surface tension on solid supports, along with contact angle values greater than 150°. Similar to superhydrophobic surfaces, superoleophobic ones find their applicability in self-cleaning, oil–water separation, controllable oil adhesion, oil caption etc. Superhydrophobic surfaces properties and applications will be accompanied by brief correlations with the superoleophobicity fast developing filed [2].

### 2.2. Superhydrophobic Surfaces’ Structure

A complete understanding of surface properties was mandatory before discovering numerous applications of superhydrophobic surfaces. The first experiments developed in this direction involved studying the structure of certain substances which confer special wettability. Thus, it has come to the hypothesis that the chemical structure of a solid support is responsible for surface heterogeneity and roughness. By manipulating these properties, one depending on the other, diverse surfaces with different properties are obtained.

In order for a surface to be called “superhydrophobic”, three conditions should be fulfilled: High apparent contact angle, low contact angle hysteresis and a high stability of the Cassie wetting state. A very interesting structure related to superhydrophobic surfaces was described by Mahadevan and Pomeau [3]. They observed that liquid droplets (water) rolled onto a hydrophobic powder bed (*Lycopodium*), result in formations called “liquid marbles”, exhibiting a superhydrophobic-like behavior.

Contrariwise, in some cases, a drop maintained stable on a hydrophobic support can be mistaken with Leidenfrost droplets which slide off a heated support, due to the so called “vapor film levitation”. It happens only as long as the support is heated over a certain temperature (the Leidenfrost temperature) and the film disappears as the stand cools. Vakarelski et al. (2012) [4] prove that superhydrophobic surface topography is important when stabilizing the vapor layer, implicitly in liquid-gas transitions on heated surfaces. The explanation lies in the fact that rugosity and porosities sustain the vapor layer, as the drop only makes contact with the rugosities’ tips. This hypothesis was adjusted, as the following demonstration was made: The contact angle attains 180° and the levitating film regime is possible at superheat temperatures. Thus, special coatings (superhydrophobic, superamphiphobic, anti-frost etc.) were developed, allowing optimal heat exchange and aqueous drag reduction.

A transition from the surface phenomena analysis, to the study of molecular interactions reveals that the strength of the hydrophobic interactions between molecules is influenced by the ionic charge. Structural modification of hydrophobic surfaces follows an optimization towards molecular recognition processes, i.e., the ability to manipulate interactions between proteins. Negative ions inserted on to hydrophobic binding sites at the antibody’s surface, generate special antibodies witch link to beta-amyloid fibrillar fragments. Thus, fibers cannot link to each other anymore, and the senile plate which induces Alzheimer’s disease no longer forms [5]. In direct correlation with cell membrane formation and protein folding, is the hydrophobic hydration phenomenon. It is also responsible for improving the hydrogen-bonding network between water molecules surrounding hydrophobic radicals. Davis et al. (2012) [6] state that the structure formed by hydrogen bonds around hydrophobic groups disappears along with the increase of temperature. The tendency of hydrophobic compounds to dispose of as “clusters” in an aqueous media is a key phenomenon, paving the way into understanding biomolecules’ dynamics.

Natural superhydrophobic surfaces exhibit hierarchical roughness at two scale ranges: Micro-and nano-roughness, as earlier presented. Hierarchical structures are unique in conferring quality superhydrophobic attributes due to nano-scaled asperities imbedded on top of micro-scaled rugosities. These rugosities stabilize the Cassie state and lower contact angle hysteresis. Experiments regarding artificial obtaining of superhydrophobic surfaces reveal that by decreasing surface roughness, the contact area between the drop and the support increases. Thus, any damage done to the hydrophobic surface affects the hydrophobicity and leads to unstable Cassie states or unwanted increasing contact angle hysteresis [7,8]. Experimental studies by Bhushan et al. [9] show that hierarchically structured plant surfaces exhibit both adhesive and non-adhesive properties. Water droplets penetrate into the micro-rugosities. Thus, they strongly adhere to the surface. Nano-rugosities are responsible for the high contact angle values. High contact angles coexist with strong adhesion to the same surface. The well-known wetting regimes: Wenzel, Cassie, Lotus and Petal may exhibit both nano- and micro-filled structures, resulting in nine wetting scenarios: Lotus, Rose petal, Rose filled microstructure, Cassie, Wenzel, Cassie filled nano- and microstructure, Wenzel filled nano- and microstructure.

Experiments by Zimmermann et al. report that superhydrophobic surfaces’ properties (performance and durability) are improved by annealing. Other investigations show how hierarchical surfaces are fabricated on silicon by etching with KOH and catalyzed etching HF/H_2_O_2_. Rugosities stand in nanostructures build on micro-pyramids. If the surface undergoes abrasion, its self-cleaning properties are reduced and hysteresis increases [7,8].

### 2.3. Superficial Energy: Empirical Models Describing Surface Phenomena

Even if superhydrophobic surfaces are ubiquitous in the environment, advanced techniques were needed to fully understand them. In order to elucidate the structure of the hierarchical surface at a micro- and nano-metric level, the research went into detail. Techniques such as: Scanning electron microscopy (SEM), transmission electron microscopy (TEM) and atomic force microscopy (AFM) were used. Thus, the obtained data along with already known surface properties, concluded that a surface is superhydrophobic if it has low surface energy and a hierarchical nano-metric structure, conferring a water contact angle greater than 150°. Young’s equation (Equation (1)) describes the equilibrium between the forces acting on a droplet placed on a solid support [1]:

(1) $$\cos\;\theta =\frac{\gamma_{\mathrm{SV}}-\gamma_{\mathrm{SL}}}{\gamma_{\mathrm{LV}}}$$

where θ is the contact angle, $\gamma_{\mathrm{SV}}$ is the solid-vapor superficial energy, $\gamma_{\mathrm{SL}}$ is the solid–liquid superficial energy and $\gamma_{\mathrm{LV}}$ is the liquid-vapor superficial energy.

**Figure 1:**
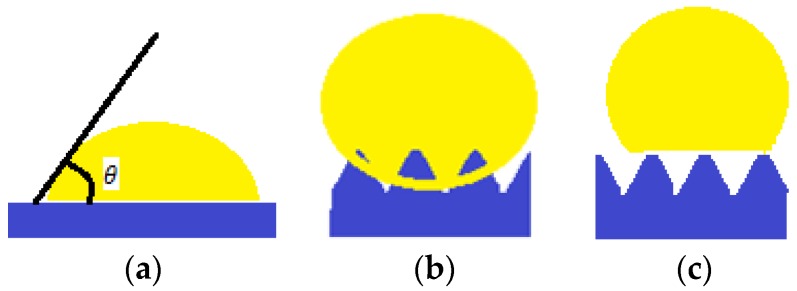
(**a**) Young wetting regime; (**b**) Wenzel wetting regime; (**c**) Cassie wetting regime.

Taking into account the fact that a minimal solid-gas superficial energy leads to a maximum contact angle, a list of surface energies for some chemical groups was established: –CH_2_– > –CH_3_ > –CF_2_–CF_2_H > –CF_3_. Nishino et al. (1999) [10], measures a minimum surface energy and a 120° contact angle corresponding to regularly arranged, close and packed groups of –CF_3_. However, Young’s equation can only be applied to smooth, homogeneous surfaces and inert to the fluid they come in contact with, as showed in Figure 1a. Following this principle, the surfaces encountered in nature do not follow Young’s wetting regime. 

In 1936, Wenzel [11] proposed an adaptation after Young’s equation (Equation (1)). The wetting regime is presented in Figure 1b. It considers a roughness factor r, defined as ratio of the actual area of the rough surface to the geometric area projected on a relatively smooth surface, and the adapted (apparent) contact angle $\theta^{\prime }$ is given by (Equation (2)):

(2) $$\cos\;\theta^{\prime }=\frac{{r(\gamma }_{\mathrm{SV}}-\gamma_{\mathrm{SL}})}{\gamma_{\mathrm{LV}}}=r\;\cos\;\theta$$

The Wenzel model applies both to hydrophobicity and to hydrophilicity, where r is a roughness measure favoring both states.

Another model describing the behavior of the liquid droplet in contact with a solid support is the Cassie–Baxter model (1944) [12]. In this case, the surface displays rugosities, and air “pockets” between them, which a liquid drop cannot penetrate, as displayed in Figure 1c.

Calculation of the adapted contact angle $\theta^{\prime }$ considers the surface in direct contact with the liquid. The fraction f is calculated as follows (Equation (3)):

(3) $$f=\frac{\sum a}{\sum \left(a+b\right)}$$

where a and b are the contact areas with the drop (a) and respectively air (b). (1 − f) stands for the drop-air contact area. Considering a contact angle of 180°, the calculation expression is (Equation (4)):

(4) $$\cos\;\theta^{\prime }=f\;\cos\;\theta +(1-f)\cos\;180^{\circ }=f\;\cos\;\theta +f-1$$

Werner’s model (2005) [13] takes into account the possibility of the drop’s penetration between the rugosities of the support. It shows a continuous increase of the contact angle, due to heavily hydrophobic “pockets” of air, which promote hydrophobicity, but only in case of rugged supports.

According to some authors, Quéré et al. (2003, 2004) [14,15] there is a critical value of the fraction f, (respectively of the critical contact angle $\theta_{c}$), under which the Cassie model can exist, and the Wenzel model is thermodynamically more stable. This state is in fact evidence that the Wenzel regime is the state of equilibrium of the Cassie model. The corresponding critical contact angle $\theta_{c}$ is determined by the following equation (Equation (5)):

(5) $$\cos\;\theta_{c}=\frac{1-f}{r-f}$$

In 2006, Yang et al. [16] develop a study with droplets placed in contact with surfaces displaying a fractal structure. It provides evidence that the contact angle depends on the average square root of surface roughness and is independent of the fractal dimension of surface $D_{f}$ at a nano-metric level.

Researches’ opinions are divided when discussing wetting states equilibrium or transitions. 

More recent studies (2017) show that both Cassie and Wenzel regimes are meta-stable and co-occur at the same surface. A “bi-stable” wetting regime is assigned to hydrophobic textured (linear, pillar) surfaces. Experiments show the following: A Cassie levitating state corresponds to drops placed onto a hydrophobic support, whilst a Wenzel “impaled” (pinned) state refers to drops after an impact with the same surface. Wetting transitions between these states were reported as being responsible for spontaneous/external stimuli (pressure, vibration) triggered changes of a drop’s contact angle. This barrier is a result of increasing liquid-air interface, as the liquid penetrates through the support. The value of the energy barrier separating Cassie and Wenzel states is attributed to a hierarchical organization i.e., surface roughness of the support. Revealing the wetting transition mechanism represents the answer in engineering highly stable superhydrophobic materials. Thus, experiments carried out by same authors reveal how wetting transitions are irreversible, due to asymmetry of the energy barrier (low from the side of the metastable state and high from the side of the stable state). Trend on future investigations are proposed [17,18].

Experiments by Yanshen et al. [18] were carried out to suppress the energy barrier, as a starter- guideline in optimizing future design of super-repellent materials. In this case, transitions between the Cassie and Wenzel state proved to be, in fact, spontaneously reversible. The method proposed by authors to probe Wenzel to Cassie (W2C) and Cassie to Wenzel (C2W) transitions implied analyzing a drop’s behavior while squeezing and releasing between a textured surface and a non-adhesive plate. C2W transitions triggered by pressure, impact, underwater submerging proved to be reversible. In addition, it was demonstrated that it is possible for the two Cassie and Wenzel wetting regimes to co-exist on a double-scaled textured surface, similar to those found in the natural environment. Thus, the Wenzel state corresponds to the larger texture and Cassie to the smaller one (nano-Cassie state). The smaller rugosities do not allow irreversible trapping of water drops. The surface in the nano-Cassie state preserves its hydrophobicity and ability to induce reversible penetration of drops through larger rugosities (slippery character). The spectacular dynamics of water drops meeting such hydrophobic/superhydrophobic textured materials remains a challenge for future investigations concerning the development of robust super-repellent materials.

Investigations concerning wetting transitions of different rough surfaces (natural and synthetic) reveal how transitions may also occur as follows: Mixed Cassie air trapping/Wenzel state to Cassie impregnating state, mixed Cassie air trapping/Wenzel state to Wenzel state, Wenzel state to Cassie impregnating state and Cassie air trapping state to Cassie impregnating state. The Cassie impregnating state is characterized by the lowest energy [19,20].

The above mentioned four wetting states (Young, Wenzel, transitions and Cassie states) also apply for an oil droplet on a flat or rough solid substrate. In this case, the corresponding liquid in the equations refers to corresponding oils [2].

In order to achieve superoleophobicity, the formation of the Cassie wetting state is crucial. Since the liquid drops exhibit low surface tension, not all rough microstructures display a Cassie wetting state. Thus, a third parameter is introduced: Re-entrant surface curvature. Along with surface microstructure and low-surface energy, it is essential in obtaining superoleophobic surfaces [2]. The importance of re-entrant surface curvature in obtaining superoleophobicity was demonstrated using POSS (polyhedral oligomeric silsequioxane) covering fiber mats. Fluorodecyl POSS displayed with oil drops contact angle smaller than 90°, whilst the covered mats showed re-entrant surface curvature and proved to be superoleophobic [21].

## 3. Special Surfaces

### 3.1. Natural Special Surfaces

Throughout history, people were captivated by the special survival skills of animals and even plants. Their close observation and study, helped human kind understand and adapt those properties into a useful approach to their evolution. Thus, following the principle of biomimetics, man built the plane after observing bird’s flight, the helicopter inspired by the body of the dragonfly, the submarine resembling a whale, and the Velcro closure system according to the way burdock (*Arctium* sp.) spreads its seeds.

Since ancient times, humans noticed the ability of plants to keep clean in marshy environments, to provide their water needs in arid areas. However, these skills remained a mystery until the 1960’s when the development of SEM analysis techniques allowed a detailed investigation of surface properties. The plants and insects that raised questions on their survival skills were studied. In 2007, it was concluded that there are two types of microstructures conferring superhydrophobicity to the leave. The first model corresponds to hierarchical micro-/nano-metric structures (Lotus, rice, taro), and the second consists of a unitary structure, an ordered fiber network, with diameters of 1–2 μm (Chinese watermelon, Ramee leaves). The idea that the surface is superhydrophobic only if it has a hierarchical structure with micronic roughness was demystified [22]. Literature brings to light leaves whose capacity to reject the water resides in the presence of vertical hairs (*Alchemilla vulgaris*) [23] or horizontal hairs (*Populus* sp.) [24].

**Figure 2:**
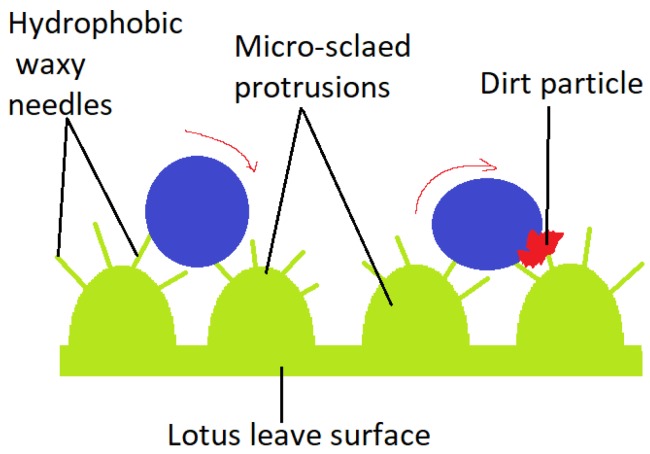
Dirt particle removed by rain drops from the Lotus leave’s surface.

An illustrative model for superhydrophobicity is the lotus leaf (*Nelumbo nucifera*), displaying a surface network which allows dust particles to be removed by rain drops [25], as illustrated in Figure 2. It’s considered to be derived from the Cassie-Baxter wetting model. At a micro-metric level, convex papillae are distinguished. At a nano-metric level, wax needles appear to be responsible for superhydrophobicity (contact angles greater than 150°). The veins placed on the top of the Tropaeolum plant leaves, secrete a wax-like substance, similar to the one on lotus leaves, providing cleansing through rolling water droplets. Curiously, the lower side of the lotus leaf has a different chemical structure and architecture, thus, inverse wettability. No waxy crystals are present, but tabular nano-grooved convex lumps cover the leaf’s lower side surface. Superoleophobicity and low oil-adhesion in water were demonstrated by a measured contact angle of 155° and sliding angle of 12.1° for a 1,2-dichloroetahne [2].

Superficial features of plants allow them to survive while floating on water, or while submerged. *Salvinia molesta* (water fern) is equipped with hydrophobic hairs which end in hydrophilic peaks [26]. They retain a layer of air, stabilize the air-water interface, while submerged under water and allow “respiration” (The Salvinia Paradox) [27]. Photosynthesis also continues in submerged *Oryza sativa* (rice), through the air film retained at the superhydrophobic leaves surface [28]. Leaf gas films enhance gas exchange, conferring plants the ability to survive during floods. They also delay salt entry in *Melilotus siculus* (melilot) by diminishing Na^+^ and Cl^−^ intrusion through the submerged leaves, allowing short-term survival in salty water [29].

Out of the plant kingdom, the carnivorous plant *Nepenthes alata* stands out as an example of a surface with special superficial properties. Experiments show that its peristome has unique structural characteristics, which allow directed water transport through a microcavity system, in the absence of any chemical gradient. The plant feeds on insects. A continuous fluid film rejects the oils from insect’s feet, sending them from the surface of the peristome into the “jug” type structure, where they are digested [30]. Many plants, such as taro (*Colocasia esculenta*), India canna (*Canna generalis baley*), rice (*Oryza sativa*), have leaves with contact angles higher than 150° and sliding angles of less than 10°, depending on the papillae arrangement [31]. Unique structures are found in *Strelitzia reginae*, which leaves are furrowed by parallel grooves. They show anisotropic superhydrophobicity, so that a drop of water remains anchored as the leaf is inclined in the grooves direction, or slides off, as the leaf leans perpendicularly to the direction of the grooves [32].

In most cases encountered in plants, a primordial importance of superhydrophobicity in leaf cleaning is conferred by the micro-modeled surfaces. There are special cases, like the *Cladonia chlorophea* lichen, in which cup shaped structures placed on hydrophobic strains, limit water storage only to the pores at the base of the stem [33]. The lichen retains only the required amount of water, banning the accumulation of any excess water, which would prevent spore spreading during reproduction.

**Figure 3:**
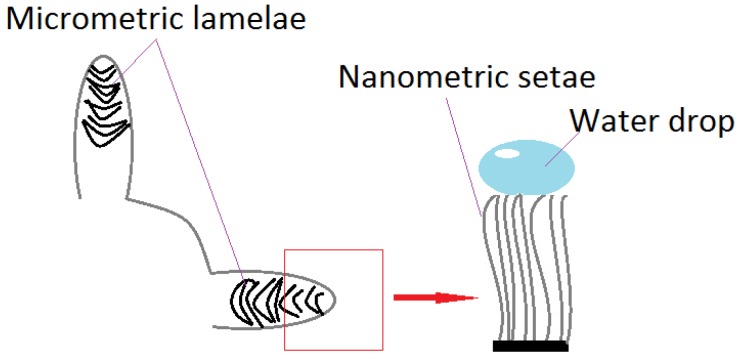
Gecko feet structure. Water drops on *setae.*

Recent research divides superhydrophobic surfaces into opposite categories, judging by interactions with a solid. Thus, there are “slippery” surfaces (Lotus leaves), which present minimum water resistance, and “adherent” surfaces (gecko lizard) [34]. It has been thought that the gecko lizard’s ability to climb, is due to structures on its fingers, which secrete adhesive substances, similar to those which allow the rise and hanging of ivy (*Hedera helix*) [35]. The lizard can climb even vertical surfaces, due to microscopic, aligned hairs, divided into nanometric formations called *setae* [36], as illustrated in Figure 3. A drop placed on this surface (a highly adhesive superhydrophobic surface) retains its shape, even in an anti-gravity position [37]. Following adhesive force’s model, climbing a glass building using Kevlar special gloves and polyurethane was accomplished (2009) [35]. The category of superhydrophobic and super-adhesive surfaces includes rose petals. They possess a network of micro- and nano-structures, similar to the lotus, but of larger size.

**Figure 4:**
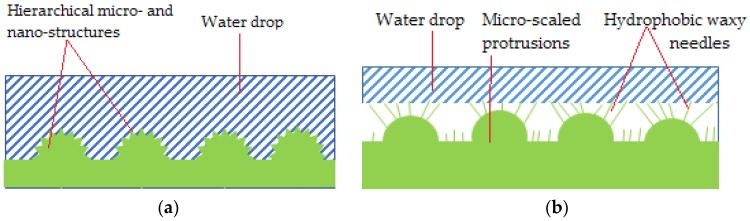
Petal surface structure (Cassie impregnating wetting state) (**a**) and Lotus leaf surface structure (Cassie state) (**b**).

The work of Jiang et al. reveal the micro-architecture of the red rose petal. Superhydrophobicity (contact angle of 152° and high hysteresis) is given by micro papillae covered in nano-folds. These rugosities make a water droplet adhere to its surface and maintain a spherical shape. Even if turned upside-down water does not fall/roll off the surface. The phenomenon is called “petal effect”, assumed to correspond to the Cassie impregnating wetting model. The Cassie impregnating wetting state is characterized by a liquid film impregnating the grooves but leaving some plateaus dry, as presented in Figure 4a. Moreover, it was demonstrated that the drop’s volume is the one conditioning this special behavior, with a different dynamic compared to the lotus effect. Thus, a small droplet sticks to the surface because its volume is smaller than the surface tension. When receding 10 µL in volume, a volume-surface tension balance is reached, and overcoming it triggers the droplet’s fall. This explanation stands up when discussing why smaller drops maintain stable spherical shape on petals and why rain drops do not and roll of [38].

When comparing the lotus leave’s hierarchical structure with the rose petal one, differences stand in microstructures and also chemical composition. Thus, a drop on a lotus leaf has a small contact angle hysteresis, waxy protrusions prevent water from entering the micrometrical structure and so the droplet is free to roll of (advancing and receding at different contact points) as the leaf is tilted. Micro and nano-structures covering the petal are both bigger than those of the lotus. That makes the drops adhere to the surface, due to water being sealed in micro papillae, while exhibiting a high hysteresis when the petal is tilted/turned upside-down [38].

Experiments were carried out in order to artificially recreate the rose petal hierarchical structure. By mimicking the rose petal effect, superhydrophobic and also adhesive polymer films were developed. The natural petal is used as a template during the fabrication process, making the method an environmental friendly one compared to other techniques [38].

Following the path of herbal substances unusual applications, Zhang (2009) [36] proposes the use of ivy nano-particles in sunscreen lotions. The additional advantage over conventional creams is that of uniform topical application, very good skin surface adhesion, and the ability to successfully block a wide range of UV radiation wavelengths. An unprecedented breakthrough, with a high impact in the medical field, is the use of tree moss to obtain adhesives. In 2014, moss was found to be non-toxic and stronger than super-glue. It proves to work successfully in wet environments, joining both soft tissues and bones [35].

Researchers interest regarding the functionality of insect wings lead to the discovery of the following: Superhydrophobicity of insect wings derives either from tiny, cape-shaped structures, miniscule hairs or from scaly formations [39]. Numerous investigated insect species reveal wings with nanometric, hierarchically disposed scale structures, which enable flight and do not allow dust contamination, as illustrated by Choi et al. [40]. Study of the cicadas, highlighted the possibility of encompassing both transparency and superhydrophobicity, by alternation of nanostructure dimensions, conferring homogeneity. Their wings are able to selectively kill Gram-negative bacteria, without attacking Gram-positive ones [41].

**Figure 5:**
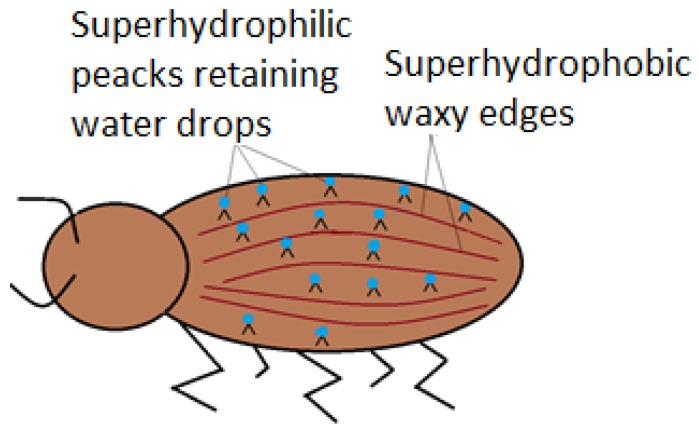
Namib desert beetle displaying superhydrophobic edges and superhydrophilic back protrusions.

Nature has its own ways when it comes to special wettable surfaces. The desert beetle (*Stenocara gracilipes*) is a special exponent of superhydrophillic surfaces joined with superhydrophobic ones. The usefulness of the association is to ensure the beetle’s need of water, in high temperature conditions. Analysis highlight that the back of the beetle is full of hydrophilic non-waxy peaks, which capture water from the fog, in form of droplets (Figure 5), as shown by Ueda and Levkin (2013) [33]. As the droplets become too large, they slip on wrinkled hydrophobic waxy edges.

Many authors [33], emphasize the importance of joining superhydrophilic surfaces (large surface energies, low contact angles ~0°) and superhydrophobic ones (low surface energies, high contact angles > 150°). Wettability variations function as advantages. Among them, the following are listed: Easy drop positioning as close as possible to each other, since the hydrophobic support does not allow interactions; simple surface geometry elaboration, including superhydrophilic water filled micro-channels. If the pattern follows the Cassie–Baxter wetting model, then the droplet’s bio-adhesion does not occur and subsequent sampling is done, keeping the contents intact. The proposed model may be a source of inspiration in creating devices capable of collecting water from fog, in arid areas.

Although the behavior of water droplets placed on Lotus leaves was studied in air, it was unknown what happens if the superhydrophobic side is turned face to water. Thus, in 2009, it was shown that droplets break as the leaf turns, showing superaerophilicity. In connection with this phenomenon, the diving spider’s (*Argyroneta aquatica*) survival underwater was explained. In order for it to breathe while submerged, it creates an artificial lung, an oxygen bubble that remains trapped between its feet and abdomen. The novelty lies in the bubble’s silk outer shell, which is hydrophobic and gas permeable, allowing underwater breathing. This approach represents a model in developing methane transport systems, in preventing undesirable underwater discharges and global warming [42].

It is important to notice chemical structure in correlation with surface architecture of the seaweed (*Saccharina japonica*). The porous structures on its surface combined with the effect of salt-insensitive polysaccharides translate into durable underwater superoleophobicity, even in high-salinity and high-ionic water. In the same wettability regime, the clam’s two region-divided shell architecture, proves to have anti-oil properties. The hydrophilic CaCO_3_ composition along with the rough hierarchical microstructure makes the shell oleophobic under water (region 2) keeping it clean all the time, whereas the other region (region 1) is polluted by oil [2].

### 3.2. Superhydrophobic Surfaces: Learning from Nature

Following the principles of biomimetics, superhydrophobic surfaces were artificially obtained. Once the surface properties (contact angle, superficial energy) of natural solids are established, they can be varied one depending on the other and applied to synthetic materials. Thus, innovative raw materials are born.

The generic method used to artificially obtain superhydrophobic surfaces follows the lotus leaf model i.e., its self-cleaning capacity conferred by wax epicuticular crystals. Its hydrophobicity arises from the –C–H and –C–O– groups [43].

Fabrication of superhydrophobic surfaces following the principle of biomimetics began in 1990. In 1992, a submicrometer-roughed glass plate was hydrophobized using fluoroalkyltrichlorosilane, with contact angles approaching 155° [44]. Among early synthetized super water repellent surfaces, the one prepared by Shibuichi et al. (1996) [45,46] included n-alkyl ketene, with contact angles of 174°, due to the fractal nature of the surface. Alumina coatings were obtained with fluorialkyltrimethoxysilane on porous alumina gel (1997). Ion-plated PTFE coatings with nano-metrical rugosities were reported in the same year [47]. Experiments were carried out by McCarthy [48] to determine the effects of topography on wettability. Patterned silicone surfaces prepared by photolithography and salinization were obtained. Their wettability was also investigated in correlation with square posts dimensions. Contact angles appeared to be independent of square posts heights and of surface chemistry, when their dimensions ranged 20–140 µm. Surfaces with square spots of 64–128 µm dimensions were not ultra-hydrophobic as expected. Water drops penetrated between the square posts and were pinned to the surface. The phenomena intensified as the distance between posts increased or as the shape of the posts was changed to rhombus, star. Thus, the maximum length scale that confers surface hydrophobicity was established at 32 µm for surfaces covered in square posts.

Ever since the first artificially obtained special surfaces were reported, various methods were improved in order to confer surface roughness, transparency, possibility of color change, reversibility, permeability, anisotropy [49,50,51]. Some methods involved the use of fluorocarbon derivatives. Nature has shown that the presence of such compounds is not mandatory in order to obtain low surface energy [43]. Techniques that produce replicas of natural surfaces were developed. Among methods used to artificially create special surfaces, depositing hierarchical micro- and nanostructures on a hydrophobic substrate or chemically modifying a low superficial energy surface are proposed by some authors [52,53,54]. 

The artificial fabricated surfaces should display a hierarchical/unitary structure. Among known procedures, the most popular are: Chemical reactions in a humid atmosphere [55,56], thermic reactions [57,58], electrochemical deposition [59], individual/layer-by-layer assembling [60,61], etching [62], chemical vapor deposition [63], polymerization reactions [64]. These techniques are applied to silicone, copper, zinc, titanium, aluminum, cotton or glass substrates, depending on the procedure. Surfaces obtained after these modifications, display contact angles greater than 150°. Among the easiest and fastest to apply techniques are hydrothermal and chemical reactions in humid air. They can be adjusted to obtain objects of any shape or size [22]. Another versatile method used to deposit a salt solution on to a metal, is electrochemical deposition, which also confers the support a furrowed structure with numerous micro-grooves [59]. Depositing carbon nanotubes onto a cotton support, creates an artificial structure similar to the lotus leaf [65]. Chemical vapor deposition proved to be efficient in “constructing” micro-pyramid like patterns with contact angles greater than 170° [66]. Sol-gel techniques which confer hexagonal ordered superficial structures may be applied on to many substrates like glass, metal, silicone, textile materials [67].

By applying the techniques previously mentioned it is possible to mimic natural special surfaces. For instance, the duplication of petals’ surfaces was achieved. Polymer films were obtained by using the red rose petal as a duplicated template. Imprinting of the nano-metric roughness is made through solvent-vapor techniques. Practically, a PVA 10% and PS 15% solutions were poured separately on different petals and evaporated. What is left behind is a PVA, respectively PS film imprinted with the micro structured pattern of the petal. The obtained films exhibit exactly the same wettability as the original petal i.e., high contact angles, hysteresis which does not allow rolling of droplets even if the plane is tilted [38].

The lotus leaf double-scaled surface pattern was also achieved. Experimental studies reveal the possibility to obtain polymeric superhydrophobic surfaces through a solvent-free ultrafine powder coating technique. Contact angles attain values of 160° and sliding angles do not exceed 5°. This method proved efficiency in mimicking the lotus leaf surface micro-and nano-scaled pattern without the use of any solvents that prove to emit toxic compounds into the environment. The method represents a breakthrough in the coating industry [68]. Large scale fabrication of special superhydrophobic surfaces is a continuously developing domain and “natural templates” still serve as models in designing innovative coatings.

### 3.3. Innovative Superhydrophobic Materials and Coatings

The previously submitted production techniques applied to obtain superhydrophobic surfaces/coatings, along with physical and chemical adsorption adjustments are applicable to non-reusable substrates (polymers, minerals).

Among low surface energy materials, fluorocarbon and silicon derivatives, some organic and inorganic compounds have been preferred for many years. Hsieh et al. (2006) [69] demonstrate that the fluorine/carbon ratio is the one influencing the superhydrophobic degree of the surface. Thus, the more fluorine atoms are included in the structure, the higher becomes the hydrophobicity. Genzer and Efimenko (2000) [70] assert that another determinant of stability and hydrophobicity is the density and layout of certain chemical groups. Experiments prove that F(CF_2_)(CH_2_)xSiCl_3_ molecules applied on a polydimethylsiloxane (PDMS) stretched substrate, immediately form an organized layer, whose contact angle increases by 30°, as the stretching force is removed.

When it comes to roughness, statistical parameters are indicative and cannot be extrapolated at a micro-/nano-metric level, for any type of surface. Geometrical roughness is considered and a direct correlation between roughness data corresponding to different sample sizes is attempted.

Polymers and minerals gained importance due to their potential as supports, with small to very small surface energies. However, these hierarchically organized structures have an inconvenient: Possible unwanted hydrophilic components. Thereby, they do not lend to the rigorous requirements of a hydrophilic barrier imposed in many fields (printing, packaging, perishable storage). In attempt to preserve the environment, these non-renewable supports were replaced with bio-based materials, derived from wood, plant fibers, agricultural residues. Lignocellulose is one example, thanks to its ease of purchase and transport, low mass and abundance. Progress has been made in the cardboard and cotton industry through producing superhydrophilic materials using cellulose as a base [1].

Following the direction of environmental-friendly materials, cellulose nano-crystals and composites receive attention. Cellulose undergoes roughening processes in order to lower its free surface energy. The structure and surface properties of cellulosic fibers are adapted through sol-gel processes or nano-particle deposition (metal oxides, minerals, polymers). Thus, the modified cellulose has both static and dynamic contact angles (hysteresis). The durability of the applied rugose layer allows any required improvements [71]. Recent experiments focused on durability and robustness of lignin-coated cellulose nano-crystal (L-CNC) particles. Commercial biodegradable L-CNC particles were used after hydrophobization (used to confer roughness). Two adhesives were used to support sticking between L-CNC particles and substrates. The resulted coatings exhibit astonishing attributes: Self-cleaning properties, water repellency, durability against sandpaper abrasion, finger-wipe, knife scratches, water jet, UV radiation, high temperature exposure, acid and alkali solution [72].

Remaining in the same field of textile materials, superhydrophobic flame-retardant cotton was developed by a researcher group using layer-by-layer assembly of branched poly(ethylamine) (bPEI), ammonium polyphosphate (APP) and F-POSS. Experiments reveal that exposed to fire, the cotton fabric and bPEI dehydrate, catalyzed by APP. Thus, a heat insulating char layer with porosities is generated. Pores are formed due to decomposition of bPEI and so the formation of flames is delayed. This is a very useful discovery for the field of flame-proof materials [73]. An effective method to use lignocellulose (LC) as base-support to fabricate superhydrophobic surfaces with flame retardant properties was recently discovered (2018). PDMS-stearic acid-modified kaolin-coated LC attains contact angles of 156°. Due to kaolin particles, it also displays good flame retardant properties [74].

Since the UV-radiations were proved to be the cause of many human health affections, UV blocking products gained popularity. Besides the well-known cosmetic products, it seems that UV blocking textile products are receiving well-deserved attention. Some studies developed UV blocking textiles using white pigments. The expansion of the green technologies reflects on the development of multifunctional textiles (self-cleaning, antibacterial). A research group demonstrated UV radiation is absorbed by PET fabric covered in ZnO/SiO_2_ pencil-shaped rods. The explanation lies in the nano-scaled rugosities and superhydrophobicity conferred by ZnO/SiO_2_, exhibiting a receding contact angle (from 160° to 90°) when exposed to UV light, due to hydrophilic groups [73]. Novel methods (2018) to produce superhydrophobic superoleophobic-covered silk textiles are reported by Aslanidou et al. Alkoxy silanes, organic fluoropolymers, silane quaternary ammonium salt and silica nanoparticles are included in an aqueous solution which is sprayed onto silk. Thus, it gains both superhydrophobicity, superoleophobicity (contact angles greater than 150°) and also antibacterial properties. The coating confers a double roughed surface architecture which also acts as an antimicrobial agent, hindering microbial growth [75].

Another way of exploiting superhydrophobicity as an advantageous property is the use of waterborne resins made of aqueous silanes and siloxanes solutions containing silica nanoparticles. Once deposited onto marble, sandstone, mortar, wood, cotton, ceramic artifacts, the composite superhydrophobic protective film turned out to be a simple, cost effective and most of all environmental friendly (no organic solvents are used) technique to protect cultural heritage [76].

Superhydrophobic self-contained surfaces, which retain their properties over time have been obtained. They represent a complete and independent unit in and of itself and are autonomous. Even so, it is difficult to mimic the self-healing properties of natural surfaces and even harder to confer wear resistance, without altering the area’s characteristics [26]. A novelty in this field is the SLIPS (slippery liquid-infused porous surfaces) technology, inspired by the lotus leaf. Basically, it refers to smooth and slippery coatings applied on sports shoes, or used in the construction field, military uniforms, medical gowns, in order to avoid biological fluid contamination. SLIPS surfaces were described as having the ability to clean themselves, due to surface fluids incorporated into the micro-/nano-porous substrate, forming a smooth surface. They are able to remove impurities with complex structures (oils, blood). No synthetic surface meets all the qualities of SLIPS surfaces: Large contact angle, lack of hysteresis, low slip angle, optical transparency, instant repair due to capillary action given by surface energy. Surfaces that serve as omniphobic materials, with possibilities to apply in fluid/biological materials, fuel transportation, glass surfaces that do not freeze and clean individually are still developing [77]. Another area in which SLIPS is being popularized is the sports footwear industry. Tests on sports shoes show that the SLIPS technology provides the best protection against moisture, but as a compromise, it does not allow the skin to be aerated. In 2009, a material whose structure mimics pine cones, with structures that open or close according to humidity was placed on the market. A single fiber includes two distinct polymers: A hydrophilic and a hydrophobic one which react according to the environmental conditions. Since 2016, another type of hybrid material has been used for yachting equipment. It is particularly useful in special activities, allowing opening and closing of temperature-dependent fibers, with the release of hot and humid air [78]. 

## 4. Applications of Superhydrophobic Surfaces in the Medical Field

As previously described, superficial properties determine superhydrophobic surfaces applications. An evolution was noticed in the use of artificial special surfaces in various fields. Continuous efforts are made in order to discover innovative means of use, in both industrial and laboratory fields, as well as in the medical field. [79].

### 4.1. Anti-Bio Adhesion

Biochemical phenomena such as protein adsorption, bacterial adhesion, and development of cell cultures are influenced by the support’s wettability, which can be manipulated in the desired direction [79]. Thus, Lampin et al. (1997) [80] experimentally demonstrate that protein adhesion is favored by hydrophobicity conferred through a PMMA (polymethyl methacrylate) coating. More recent studies by Zelzer et al. (2008) [81], show that fibroblasts adhere differentially to a surface exhibiting a chemical gradient: From hydrophobic (polymerized hexane plasma) to hydrophilic (allylamine-polymerized plasma). In order to minimize activation and adhesion of blood platelets on to an implant/prosthesis surface, in vitro experiments have been carried out. It was demonstrated that platelets do not adhere or propagate further, on supports covered in TiO_2_ nanotubes matrices, which are compatible with blood. In 2009 [82], four types of PDMS (polydimethylsiloxane) surfaces with rugosities of various sizes were developed: Superposed scale plates, sub-micron structures, nano-structured and smooth surfaces. The superposed scale plate surface proves to be the most effective against adhesion of blood platelets, under biological blood flow conditions. Statistical results also show a low surface adhesion, whilst the highest degree of adhesion corresponds to the smooth surface.

### 4.2. Anti-Bacterial Fabrics

Surface roughness combined with a low superficial energy, led to the artificial production of surfaces exhibiting special wettabilities, i.e., superhydrophobic surfaces. Considering liquids with a superficial energy lower than water (decan, octane [21], oils) and taking into account an additional parameter, surface curvature, superoleophobic surfaces with contact angles greater than 150° were developed [2]. Superoleophobic cellulose fibers have been obtained by modifying siloxane with silver nanoparticles, with the help of an active organic/inorganic binder. The inherent property of these fibers resides in antibacterial activity against *Escherichia coli* (100% suppression rate) and *Staphylococcus aureus*. Additional treatments applied to cotton fabric improved antibacterial activity, conferring durability and ease of washing [83]. Through special treatments with fluoroalkyl silanes, glass acquires its antibacterial action against small concentrations of *Pseudomonas aeruginosa* and *Staphylococcus aureus*. Aizenberg et al. [84] show that PEG films exhibit much lower antibacterial activity compared to fluorinated silicone oil infused slippery surfaces capable of removing 96–99% of the bacteria. Special coated (Siloxane + antimicrobial agent (am) + SiO_2_) superhydrophobic silk shows a decrease in spore adhesion and growth compared to uncoated or Siloxane + am agent coated samples, kept in the same conditions [76].

### 4.3. Cancer Cell Isolation

All cell types (including cancerous cells) display certain protrusions on the membrane’s surface, through which they interact with the biological medium: Bind to target cells/tissues or reject possible harmful agents (macrophages, antibodies). Thus, any medical material/implant/device that comes in contact with the human body, should be carefully designed. Attached cytophilic/cytophobic moieties (superhydrophilic/superhydrophobic), influence detection of circulating cancer cells [85,86]. Nanometrical printed roughness confers superhydrophobicity, captures only cancerous cells and does not allow adhesion of healthy blood cells [42]. Improvements have been made so that nanostructures–cell interactions successfully remove ill cells from the patient’s blood. This method opens new gates in early diagnosis of rare cells which cannot be done using existing technologies (biopsy) [87]. Galactozylated carbon nanotubes are also cytotoxic structures, and can be used to capture viruses or bacteria [88].

## 5. Special Patterns. Joining both Superhydrophobic and Superhydrophilic Surfaces

**Figure 6:**
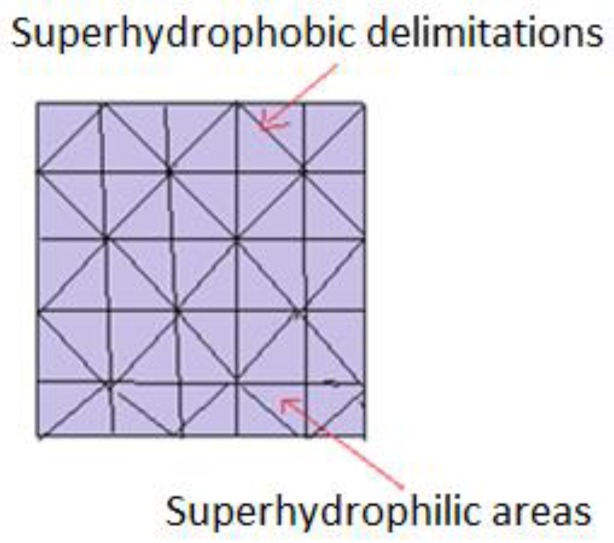
Schematic representation of patterns joining superhydrophobic with superhydrophilic areas.

In order to find more effective methods to study biomolecules’ (peptides, oligonucleotides, enzymes) activity, innovative techniques have been developed at a small scale i.e., micro-pattern supports, made from superhydrophobic and superhydrophilic adjacent areas, as illustrated in Figure 6. Basically, molecules of interest, in the form of aqueous solutions, are deposited on hydrophilic areas. They remain separated from each other, due to hydrophobic zones which surround them, which do not allow migration or mixing. A single support may comprise hundreds of droplets, with volumes ranging from pico to microliters. Another proposed model implies that pre-impregnated spots carry cells of interest, selected from a library of molecules. Biomolecules are included both in aqueous solutions and isolated hydrogels, allowing 3D screening and complete immersion in a common environment. Comparative analysis of cell behavior and creation of a supportive culture medium is carried out. Microscopic analysis can be achieved due to the transparency of hydrophilic spots, separated by opaque superhydrophobic barriers [34,89,90].

### 5.1. Peptides Separation

Wettability can also be set up as a grading criterion. Thus, 2D thin layer chromatography techniques are applied to separate peptides with different hydrophilicities and isoelectric points. Separation happens due to a superhydrophobic porous polymeric support, engraved with superhydrophilic channels. In fact, there are two processes that lead to separation: One inside the microchannels, as the aqueous mobile phase migrates to the hydrophilic areas, guided by the superhydrophobic pattern on support; the second process is based on the use of a mobile phase with acetonitrile (the separation itself). The separation occurs according to the hydrophobicity of the peptides. Detection is made through desorption or electrospray ionization. This method gives rise to the use of micron-sized diagnostic systems, by joining superhydrophobic patterns with superhydrophilic ones [91].

### 5.2. Molecules Screening

Hydrophobic/hydrophilic merged areas were developed at a micrometric level, in order to synthesize new inhibitors of serine protease NS3/4A, a promoter of hepatitis C virus. The pattern consists of areas with hydrophilic points, which ensure the stability of nano-droplets, placed on a hydrophobic support. NS3/4A inhibitors were synthesized into the drops [92]. Microdroplets placed on these special surfaces, allowed analysis of auto-fluorescent molecules, with possible applications in non-invasive diagnosis and real-time imaging [93].

Techniques based on merging different wettability surfaces, proved to be useful in assessing molecular and enzymatic kinetics, completing studies on drugs’ mechanisms. The advantages of these techniques include: The possibility to control pattern’s geometry, droplets position and volumes, safety by means of droplets stability which cannot migrate to another adjacent formation, ability to easily handle small volume drops, space and reagent saving, as well as fast by means of preparation and analysis methods [89].

## 6. Applications Derived from Water’s Behavior

### 6.1. Anti-Icing Properties

The optimal functioning of airplanes, boats, telecommunication routes and highways is influenced by ice formation. Over the years, procedures were developed to avoid the frost of these surfaces. In recent years, the use of superhydrophobic materials that prevent/reduce condensation and ice formation became popular [42].

The anti-freeze property of superhydrophobic surfaces is already well known. Liangliang Cao et al. (2009) [94] discover a correlation between particle size and anti-freeze properties. An important aspect is brought to light: There is a difference between the particle size which confers superhydrophobicity and the one conferring anti-freeze characteristics. Obtaining a surface with both attributes has been a challenge in terms of factors influencing water’s frost on a surface: The adhesion of ice on superhydrophobic surfaces and environmental conditions. The study is based on including a polymeric binder (silicone resin, acrylic polymer, silica particles) in the composition of nano-particulate polymeric surfaces. Further research is needed to establish a superhydrophobic surface design that satisfies the rigors of anti-freeze surfaces [94]. It is desirable to develop coatings which maintain dryness of the support, determining the drops to bounce off the surface. This way, metal rusting and airplane wings frosting can be avoided [26]. Attempts were made to link surface frost to the “ascending/descending” contact angle observed in ice adhesion on steel surfaces. Thus, by increasing the “withdrawal” contact angle, ice adhesion intensifies [95]. Two-leveled highly hydrophobic surfaces which do not allow formation of ice crystals, not even at extreme temperatures (−30 °C) were developed [96].

In 2013, experiments were conducted to analyze the behavior of a droplet falling on a fluorosilane-coated surface. Within milliseconds, the following events take place: Surface scattering, kickback, lifting. The events start with the drop’s impact, which then stimulates the center’s lift and the entire drop rebound [97]. In 2015, the droplet’s jump off the superhydrophobic support was elucidated through an experiment developed in a dry-aired room and under low pressure. The drop rests on the rugosities of the support. Beneath it, are air voids including water vapors. Here the pressure is higher compared to the environmental one, resulting in the droplet’s lifting. Another explanation is based on the sudden freezing of the already cooled droplet, which causes a rise in pressure and jump of the droplet. Of course, methods involving lowering ambient pressure cannot be applied to avoid frost in open spaces, but can be used at laboratory level [98].

### 6.2. Oil–Water Separation 

Industrial accidents and massive spills resulted in enormous quantities of ecosystem-damaging oils and mixtures being discharged in seas and oceans. In order to support water cleaning, systems based on superhydrophobic/superoleophilic materials are developed [42].

Meshes of porous materials superhydrophobic–superoleophilicity or superoleophobicity–superhydrophilicity were a success as oil–water separation devices. An oil removing mesh removes oil form a water-oil mixture. Water did not wet the mesh due to the superhydrophobicity, but the oil fully wetted and permeated due to superoleophilicity. Separation occurs successfully. The only issue stands in the fact that oil blocks the mesh, decreasing the efficiency of the separating material. Other meshes, able to remove most oils (with lower density than water) were developed [2]. Diesel can be separated from water using Teflon-coated stainless-steel mesh systems. Organic solvents can be separated by absorption through nano-fiber membrane systems, which combine superhydrophobicity/superoleophobicity with capillarity [99]. In addition, by combining the two surface properties and adapting them within an aqueous medium, a net covered in a hydrogel was obtained. It separates water from crude oil, gasoline, and diesel oil. It is easy to clean and can be reused, putting an end to the waste water pollution process [100].

## 7. Applications of Superhydrophobicity in Other Domains

Nowadays, as our planet becomes more polluted, self-cleaning and anti-fouling materials are of much need. By means of self-cleaning, many superhydrophobic applications are included, such as anti-bio adhesion, as earlier presented. In addition, anti-reflective, anti-icing/fogging materials, water purification systems are of high demand when it comes to extreme situations (industrial oil spills, foggy airplane windows etc.) [36].

### 7.1. Self-Cleaning Textiles

Most self-cleaning surfaces exhibit a contact angle greater than 160° and an architecture similar to the lotus leaf. Multi scale roughness and low energy waxes are responsible for the superhydrophobic and self-cleaning property of artificially made surfaces which mimic the lotus leaf pattern. Recent progress in developing such surfaces rely on two appropriate yet different techniques: Constructing hierarchical rugosities on a hydrophobic surface and coating a rough surface with a low energy material. Windshields, windows, building or ship paintings, solar panels benefit upon the possibility of drag reduction, lower sliding angles [101].

As a drop rolls off a hydrophobic support, dirt particles are displaced to the drop’s sides and re-deposited as the liquid slides off. Superhydrophobic surfaces allow the drop to easily slide off their surface and also gather solid unwanted particles. A low adhesion degree corresponds to the so called “self-cleaning” property [35]. The most illustrative examples are the previously presented lotus leaves and rose petals.

Textiles and materials with such self-cleaning properties are desirable. Thus, emerging procedures are applied to textiles/surfaces in order to confer water, oil, dirt-repellency. Techniques are still being developed in terms of cost-effectiveness, durability by means of extreme temperature exposure, wash resistance. Among popular methods, sol-gel processes, using fluoroalkyl water born siloxane (FAC), silver nanoparticles and inorganic binders prove effectiveness in preventing adhesion and growth of bacteria. The “plasma” technique, compared to classical chemical methods, offers the simultaneous advantage of roughness and low surface energy. After applying this technique, the material undergoes structural changes, gains nano-scale roughness and preserves its color and texture. Experiments by Vasiliević et al. regarding modifications of cellulose surface in order to induce superhydrophobicity, oleophobicity confirmed that a cotton fabric surface can behave similar to the lotus leave due to low-pressure water vapor plasma pre- treatment followed by the addition of a sol-gel coating using FAS precursor. FAS coating proved to offer superhydrophobicity to the cotton fiber and the plasma pre-treatment prior to that coating provided water and oil repellent properties. The plasma pre-treating process does not ensure durability of the lotus effect during repeated washing processes but enhances the formation of a FAS concentrated coating network [102]. Other studies by the same authors were made to establish the influence of oxygen plasma treatment on the water repellency of cotton fibers coated with an inorganic-organic hybrid sol-gel perfluoroalkil-functionaized polyselsequioxane (SiF). Regardless of the applied time and opperating current, the oxygen plasma treated fabric experienced an increase in contact angle value from 135° to 150°. It became obvious that plasma treatment influenced the water repellency induced by the SiF coating. The resulted cotton surface gained rugosities. It can be concluded that the plasma pre-treated and SiF-coated cotton fabric attaines micro- and nano-asperities which strongly determine hydrophobicity [103].

Xi Yao et al. developed a technique that produces superamphiphobic cashmere textiles (superhydrophobic and superoleophobic). Even more, the above presented techniques are economical and weakly polluting processes, that can be applied at an industrial level [79].

Some authors attain the self-cleaning property by modifying textile surfaces loaded with hydrophilic TiO_2_. The surface also exhibits an antibacterial effect (due to Ag deposited on the activated cotton). The interest in using such bactericide/antifungal/antiviral textiles resides in the necessity to use topical treatments of skin diseases [104].

Self-cleaning properties of silk were achieved also by covering it in siloxane enriched with 2% SiO_2_ nanoparticles. Any liquid is absorbed by the untreated silk. Superhydrophobicity and superoleophobicity are obtained. The wax is easily removed mechanically from the treated surface leaving behind no residue. On the untreated silk, a stain remains after wax removal. Durability of the coatings over a wide range of pH is demonstrated: Surfaces maintained their properties except for pH basic conditions. Additionally, a recent “green cleaning method” is presented using supercritical CO_2_ at bar and 40 °C. Dirt is removed without affecting natural dyes. An equally important aspect is the fact that siloxane and SiO_2_ coatings do not affect appearance of silk. Since no organic solvents are used, the coating method is friendly to the user and the environment. Moreover, the coating technique is reversible, allowing removal from the silk substrate using compressed CO_2_ mixed with methanol [105].

Unlike superhydrophobic surfaces, whose cleaning properties rely on the Lotus effect i.e., the dust is collected through water drops rolling off the leaf function, superamphiphobic surfaces can be kept clean by droplets, which during the rolling process, adhere to the surface and thus remove dirt particles. Underwater superoleophobic materials with ultralow oil-adhesion also exhibit self-cleaning properties. The oil adheres to the surface. After immersion it is completely removed from the surface. Self-cleaning effects underwater for superoleophobic surfaces is due to the intrinsic superhydrophilicity. Through immersion, water is injected into the microstructures and oil is pushed out [2].

### 7.2. Anti-Reflective Transparent Coatings

Glass used to fabricate mirrors, lenses, optical devices, exterior windows and solar panels should display self-cleaning abilities. Apart from self-cleaning, superhydrophobic surfaces can be exploited by means of anti-reflective properties. A special interest is also attributed to transparency and the ability not to reflect light beams. These characteristics can be conferred to glass by intervention upon roughness, a property closely related to transparency. Rugosities of nanometric dimensions are conferred by silicon derivatives (fluoroalkyl silanes), aluminum acetyl acetonate [106,107,108]. A surface film is formed, the thickness of which can be adjusted according to the desired degree of transparency. The surface film may be multilayered, each layer having its own adjustable characteristics. Polymeric films are the most flexible and resistant compared to inorganic ones [109].

### 7.3. Corrosion-Resistant Metals

Metal corrosion is one of the contemporary problems faced by humanity, which leads to large losses of money because of damaged areas, which require later replacement. Coating processes with chromium derivatives protect metallic corrosion surfaces, but they are environmentally harmful methods, which tend to disappear. New corrosion protection techniques are being sought, one of them being: Metal coating with superhydrophobic protective layers [110]. Between the protective layers and the support, an air cushion is created, thus preventing penetration of the corrosive agents. Differential coating techniques (microwave chemical vapor deposition, followed by immersion) with fluorochloride-silanes of magnesium alloys are used. Stable corrosion resistant coatings have been obtained, which reveal a color change from silver to green [111,112]. Subsequent studies refer to superhydrophobic aluminum substrates modified with hydroxides, zinc immersed in superhydrophobic solutions, proving resistance against acids, alkaline or saline solutions [113].

### 7.4. Microreactors

When studying chemical reactions, rationalization of reagents and limitation of secondary explosive/toxic compounds is primordial. Minimization of the entire chemical reaction scale starts with modifications applied to the reaction medium, reactants and by-products [114]. Microemulsions and microfluidic systems gained attention through their ability to miniaturize the chemical reaction, at levels corresponding to nano- or micro-liters [115].

While handled with superhydrophobic tweezers, liquid droplets placed on a non-adhesive support, constitute a reaction medium/reagent. The drops retain their shape, do not lose components, and can even be transported using a nano-particle composite wrapped tweezers. By forcing the drops together, their coalescence takes place. The resulting droplet functions as a “reaction plant”. Two components from different droplets react, giving rise to a new reaction product. The advantages of the method are: The possibility to obtain new, fully collectable compounds in small amounts and by using minimal quantities of reactants, in a controlled environment. The resulted new products can be detected due to color change (i.e., yellow becomes colorless as the coalescence between a drop of tetrachloromethane bromine and styrene tetrachloromethane occurs) and sampled using tweezers [42]. Decomposition, etherification, combining and controlled temperature reactions can occur. A drop of water in oil can accommodate a reaction produced by heating over time, without the aqueous phase’s evaporation. Restriction of experimental chemical reactions on a micrometric scale finds its applications in DNA analysis, synthesis of new molecules, new active substances, and may constitute basics of innovative analysis methods [116,117]. It is noteworthy that microdroplet manipulation was achieved through an oil-based microreactor, which relies on the controllable oil-adhesive superoleophobic surfaces. The droplet-based microreactors are important in enzymatic kinetics, protein crystallization processes as alternative controlled transporters to expensive micropumps, microvalves, microchannels [2].

### 7.5. Friction Reduction

The field of aeronautics and water transportation (ships, submarines) are governed by the unwanted phenomenon of water and air friction [118]. Removal strategies for this undesirable factor include development of surfaces with special structures inspired by the shark skin or Lotus leaf pattern. Experiments show that superhydrophobic surfaces have the ability to reduce fluid friction, both in laminar and turbulent flow. Air bubbles embedded between the rugosities (responsible for superhydrophobicity), result in a continuous surface film, thus reducing friction. The problem arises in the fact that the film disappears under high pressure. Recently, a hydrophobic pattern support, whose gaseous film is stable in extreme conditions was created [42].

### 7.6. Novel Transportation Devices

Transportation means invented by humans are inspired by nature (birds, insects, whales). The same applies in case of the water strider’s needle-like rugosities, which confer superhidrophobicity to the spider’s feet, allowing it to walk on the water surface. These insect’s special features represent a starting point in developing prototypes of miniature robots similar in appearance and structure. Having the ability to move in a straight line and jump at the water’s surface, while collecting information on its composition, these robots are used to monitor environmental water. Other models of robots have feet made of nanoparticles of organic semiconductors. They display high transport capacities in relation to their own dimensions. These suggestions materialize in models for developing innovative aquatic transport devices [119,120].

Micrometrical droplet transportation devices rely on superoleophobic surfaces exhibiting low oil-adhesion, which act as anti-oil “agents”. If the oil-adhesion on a such surface is high, then the oil sticks to the surface, even if tilted. This may constitute a micro-oil-droplet transportation device [2].

Passing from transportation means on water, to water transportation itself, it comes to the idea that liquids can be transported by simply modifying surface’s wettability. The roughness of the surface determines wettability, directing the movements of the water droplet. In addition, the intervention of external stimuli (light, electric current, magnetic field) can intervene and guide the fluid on the support surface [121,122]. By adjusting rugosities according to needs, the adhesion degree of the support can be controlled. An illustrative example of these assumptions is the lossless transport of micro-particulate water on superhydrophobic surfaces with PS nano-tubes [79].

### 7.7. Water Storage

Hydrophilic prominences alternating with superhydrophobic channels, make up the Namib beetle’s chitin pattern. It represents an inspirational model for researchers in developing water collecting devices [123]. The alternation between different wettability areas, determines water collection. Various arrangements (circular, vertical, horizontal), can be adopted, depending on the case. Experiments show that droplet volume does not influence the force keeping the droplet anchored to the surface. Studies are being carried out to optimize these surfaces, through the contrast between surface wettability [42]. Promising applications spring from these patterns and refer to the ability of capturing water from the fog in desert areas, to save fresh water during drought periods.

### 7.8. Electronic Components

Surface characteristics have been explored in order to obtain small electronic components such as electrodes, inductors, transistors. They have textured surfaces with varying degrees of wettability. Obtaining methods include inkjet printing of surfaces with hydrophobic regions, which reject ink, channeling it to hydrophilic regions, thereby producing self-aligned, printed patterns [124].

## 8. Conclusions

This paper concentrates interdisciplinary researches on surface wettability, bringing together studies on superhydrophobic special surfaces. It focuses on structure, means of obtaining, respectively on practical applications of superhydrophobic surfaces, starting from models existing in the natural environment (lotus leaf, butterfly wings, etc.). Following the principle of biomimetics, researchers developed systems that allow 3D screening of biomolecules, isolation of cancer cells, self-cleaning textiles to prevent biological fluid adhesion. In addition to high-impact medical discoveries, systems being able to analyze the chemical composition of water were also fabricated. Superficial properties of superhydrophobic surfaces allowed the micrometric study of chemical reactions (microreactors) using ecological and economical techniques. All these researches have a common direction: Elaboration of efficient, fast and economical methods, applicable at an industrial level, in order to obtain special wettable surfaces or protective coatings. Exploration of the advantageous features of surfaces with special superficial properties continues.
